# Novel homologous lactate transporter improves l-lactic acid production from glycerol in recombinant strains of *Pichia pastoris*

**DOI:** 10.1186/s12934-016-0557-9

**Published:** 2016-09-15

**Authors:** Pollyne Borborema Almeida de Lima, Kelly Cristina Leite Mulder, Nadiele Tamires Moreira Melo, Lucas Silva Carvalho, Gisele Soares Menino, Eduardo Mulinari, Virgilio H. de Castro, Thaila F. dos Reis, Gustavo Henrique Goldman, Beatriz Simas Magalhães, Nádia Skorupa Parachin

**Affiliations:** 1 Grupo de Engenharia Metabólica Aplicada a Bioprocessos, Instituto de Ciências Biológicas, Universidade de Brasília, Brasília, DF CEP 70.790-900 Brazil; 2 Pós-Graduação em Ciências Genômicas e Biotecnologia, Universidade Católica de Brasília, Brasília, DF CEP 70.790-160 Brazil; 3 Faculdade de Ciências Farmacêuticas de Ribeirão Preto, Universidade de São Paulo, São Paulo, CEP 14.040-903 Brazil; 4 Integra Bioprocessos e Análises, Campus Universitário Darcy Ribeiro, Edifício CDT, Sala AT-36/37, Brasília, DF CEP 70.904-970 Brazil

**Keywords:** l-Lactic acid, *Pichia* (*Komagataella*) *pastoris*, Lactate transporter, Oxygen limited fermentation, Lactate dehydrogenase

## Abstract

**Background:**

Crude glycerol is the main byproduct of the biodiesel industry. Although it can have different applications, its purification is costly. Therefore, in this study a biotechnological route has been proposed for further utilization of crude glycerol in the fermentative production of lactic acid. This acid is largely utilized in food, pharmaceutical, textile, and chemical industries, making it the hydroxycarboxylic acid with the highest market potential worldwide. Currently, industrial production of lactic acid is done mainly using sugar as the substrate. Thus here, for the first time, *Pichia pastoris* has been engineered for heterologous l-lactic acid production using glycerol as a single carbon source. For that, the *Bos taurus* lactate dehydrogenase gene was introduced into *P. pastoris*. Moreover, a heterologous and a novel homologous lactate transporter have been evaluated for l-lactic acid production.

**Results:**

Batch fermentation of the *P. pastoris* X-33 strain producing *LDHb* allowed for lactic acid production in this yeast. Although *P. pastoris* is known for its respiratory metabolism, batch fermentations were performed with different oxygenation levels, indicating that lower oxygen availability increased lactic acid production by 20 %, pushing the yeast towards a fermentative metabolism. Furthermore, a newly putative lactate transporter from *P. pastoris* named PAS has been identified by search similarity with the lactate transporter from *Saccharomyces cerevisiae* Jen1p. Both heterologous and homologous transporters, Jen1p and PAS, were evaluated in one strain already containing LDH activity. Fed-batch experiments of *P. pastoris* strains carrying the lactate transporter were performed with the batch phase at aerobic conditions followed by an aerobic oxygen-limited phase where production of lactic acid was favored. The results showed that the strain containing PAS presented the highest lactic acid titer, reaching a yield of approximately 0.7 g/g.

**Conclusions:**

We showed that *P. pastoris* has a great potential as a fermentative organism for producing l-lactic acid using glycerol as the carbon source at limited oxygenation conditions (below 0.05 % DO in the bioreactor). The best strain had both the LDHb and the homologous lactate transporter encoding genes expressed, and reached a titer 1.5 times higher than the strain with the *S. cerevisiae* transporter. Finally, it was also shown that increased lactic acid production was concomitant to reduction of acetic acid formation by half.

## Background

Bio-based chemical production from renewable sources has received considerable attention in recent decades due to both economic and environmental concerns, such as the price of petroleum-derived compounds and the effect of residue accumulation on the Earth [1]. Therefore, biotechnological routes using raw materials and renewable sources for the production of bulk chemicals have been studied as an important alternative to conventional petroleum-based processes [2, 3].

Among available raw materials, crude glycerol has gained attention in recent years for being the main waste product in the conversion of vegetable oils into biodiesel. Brazil is the second largest biodiesel producer reaching a production capacity in May 2016 of 19,976.81 m^3^/day of pure biodiesel, resulting in approximately 1997.7 m^3^/day of crude glycerol [4]. Therefore, with the aim of developing strains that will further be able to use crude glycerol as a carbon source, glycerol was used in this study as the substrate for genetically engineered *Pichia pastoris* strains to produce lactic acid. Recent studies have reported metabolically-engineered microorganisms such as *Escherichia coli* [5, 6], *Rhyzopus oryzae* [7] and *Enterococcus faecalis* [8] for improving lactic acid (2-hydroxyproponoic acid) production using glycerol as a single carbon source. However, bacteria have to overcome low tolerances toward acidity when producing lactic acid, while yeasts are generally resistant to low pHs. In addition, yeasts are generally robust and resistant microorganisms which can survive in industrial conditions, and are thus easy to use in scale-up bioprocesses [9].

Lactic acid is an organic acid commonly produced by diverse organisms such as bacteria (e.g. *Corynebacterium glutamicum* and *Bacillus* strains), fungi (e.g. genus *Rhizopus*), yeasts (*Saccharomyces* and *Kluyveromyces* genera), and microalgae (e.g. *Scenedesmus obliquus*) [10]. It is currently utilized in food, pharmaceutical, textile, leather, and chemical industries, making it the hydroxycarboxylic acid with the highest market potential worldwide [11]. Moreover, lactic acid is the monomer used for the production of biodegradable poly-lactic acid (PLA) that can be used in automobile, packaging and cosmetic industries [10]. PLA is essentially produced by the direct polymerization of lactic acid, and has its physical and mechanical properties determined by the purity of the two lactic acid optical isomers, l- and d-lactic acid [12]. Therefore, depending on the characteristics of the desired PLA, both isoforms have to be produced independently so they can be used in correct proportions.

The methilotrophic yeast *Pichia pastoris* naturally grows in high densities on different carbon sources such as glucose, glycerol, methanol, and sugar alcohols [13]. On account of its ability to grow on defined medium achieving high cell densities and its preference for respiratory mode, decreasing the excretion of by-products like acetate and ethanol, this system is a powerful candidate for utilization at the industrial scale [14]. Moreover, methanol, the primary contaminant of crude glycerol, has no negative impact on *P. pastoris* growth [15]. In fact, this microorganism has higher biomass production growing in crude glycerol than in pure glycerol, indicating that *P. pastoris* can even utilize the contaminants from the biodiesel transesterification process for biomass formation [16].

In this study, for the first time, the *LDH* encoding gene of lactate dehydrogenase from *Bos taurus* was cloned under the control of the GAP constitutive promoter and introduced into *P. pastoris* strains. Nevertheless, the obtained yield was only 10 % of what is theoretically possible. In order to evaluate whether l-lactic acid production could be improved, different oxygenation conditions were tested. Moreover, two lactate transporter coding-genes were also evaluated: the lactate transporter Jen1p from *Saccharomyces cerevisiae* and the putative *P. pastoris* lactate transporter, identified for the first time in the present work. All constructed strains were evaluated in fed-batch experiments for glycerol consumption and lactic acid production. The best strain containing both LDH and Lactate transporter activity reached 70 % of yield.

## Results

### l-lactic acid production in *P. pastoris*

The codon-optimized *ldh* encoding-gene from *B. taurus* was introduced into the *P. pastoris* genome by homologous recombination. The selected colonies were grown in selective medium and were used to measure LDH-specific activity. All colonies showed LDH activity with statistical differences compared to the wild-type strain, however, one clone demonstrated higher activity among the selected clones (data not shown), here named XL (Table 1). In order to test the physiological behavior of XL, batch fermentation containing 4 % glycerol was performed and proved its ability to convert glycerol into lactic acid, confirming heterologous LDH production [17].

**Table 1:** Plasmids and strains used in this work

Plasmids/strains	Genotype	Reference
*Plasmids*		
pGAPZB	*Pichia* integrative plasmid; Zeocin^®^ selection; Expression controlled by constitutve GAP promoter	Invitrogen^®^
pPICPGKGFP	Green Fluorescente enconding gene cloned under the controlo f PGK promoter	Personal communication
pGAP-LDH	LDH^+^, *Bos taurus* gene encoding for LDH enzyme	This work
pPGK-JEN	JEN1^+^, *S. cerevisiae* gene-encoding for the lactate transporter Jen1p	This work
pPGK-PAS	PAS^+^, *P. pastoris* gene-encoding for a putative lactate transporter PAS	This work
*E. coli strains*		
DH5α™	F– Φ80*lac*ZΔM15 Δ(*lac*ZYA-*arg*F) U169 *rec*A1 *end*A1 *hsd*R17 (rK–, mK +) *pho*A *sup*E44 λ– *thi*-1 *gyr*A96 *rel*A1	Life technology
DH10B™	F– *mcr*A Δ(*mrr*-*hsd*RMS-*mcr*BC) Φ80*lac*ZΔM15Δ*lac*X74 *rec*A1 *end*A1 *ara*D139 Δ(*ara leu*) 7697 *gal*U *gal*K *rps*L *nup*G λ–	Life technology
*P. pastoris* strains		
X-33	Wild type	Life technology
XL	X-33 + pGAP-LDH	
GS115	*his4* ^−^	Life technology
GJ	*his*4^−^ + pPGK-JEN	This work
GLJ	*his*4^−^ + pGAP-LDH + pPGK-JEN	This work
GS	*his*4^−^ + pPGK-PAS	This work
GLS	*his*4^−^ + pGAP-LDH + pPGK-PAS	This work

**Fig. 1:**
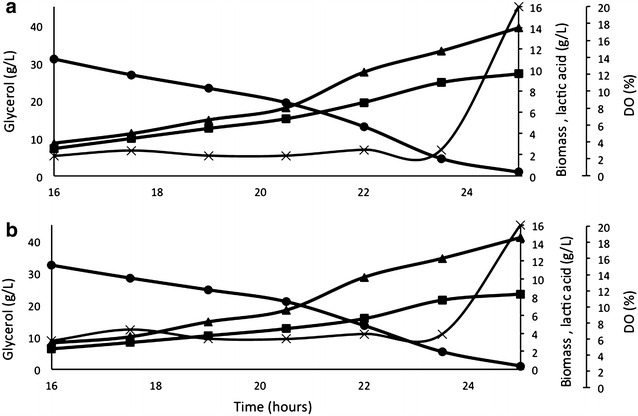
XL batch fermentation using glycerol (*filled circle*) as carbon source and limited dissolved oxygen supply by 3 % (**a**) and 5 % (**b**) for the production of biomass (*filled triangle*) and lactate (*filled square*). Experiments were performed in triplicate. The figures show the profile of one of the fermentation within 10 % error

*Pichia pastoris* is an obligate aerobe yeast [18]. In order to evaluate whether oxygen limitation would improve lactic acid production, batch cultivations were performed at two different dissolved oxygen levels, 3 and 5 %. When supplied with 3 % dissolved oxygen, the XL strain had a lactate yield (lac/s) of 0.236 g/g, and when supplied with 5 % dissolved oxygen, it had 20 % lower yield (Y_lac/s_ of 0.196 g/g). Moreover, XL µmax at 3 % was about 10 % lower (0.174) than at 5 % (0.189) indicating a higher conversion of glycerol into biomass when higher amounts of dissolved oxygen are supplied, lowering the production of lactic acid (Fig. 1).

### Identification of a putative lactate transporter in *P. pastoris* and ^14^C lactate transport assay

In *S. cerevisiae,* the activity for the lactate–proton symporter has been described to be dependent on JEN1 gene expression [19]. Expression of *S. cerevisiae* Jen1p lactate transporter in reconstituted heterologous *P. pastoris* membrane vesicles demonstrated that Jen1p is a functional transporter [20]. A BLAST search (http://www.blast.ncbi.nlm.nih.gov/blast.cgi) aiming to identify the *jen1* orthologous gene in the *P. pastoris* genome was performed and revealed an open reading frame with significant similarity (50.19 % identity). The protein of the putative homologue, PAS, was predicted to have 552 amino acids in length and to belong to the proton-linked monocarboxylate transporter family (accession number: XM_002492622.1). The Jen1p proteins contained 12 of the predicted transmembrane segments, while PAS contained only 10 helices.

**Fig. 2:**
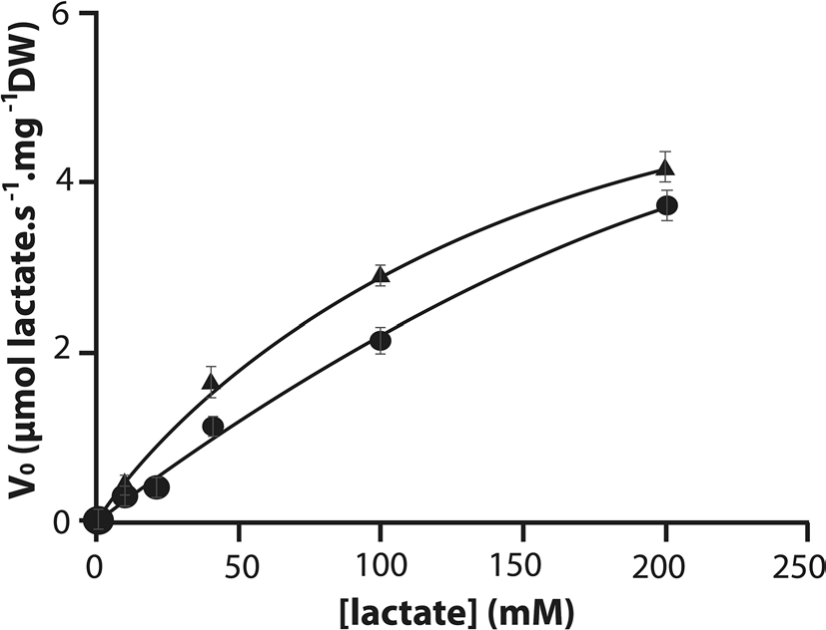
Uptake rates of labeled lactic acid measured in GS115- (*filled circle*) and GS (*filled triangle*) strains. Experiments performed in triplicate

Subsequently, the *PAS* gene was inserted into the *P. pastoris* GS115 strain under the control of the phosphoglycerate kinase 1 promoter, resulting in the GS strain (Table 1). Similarly, the *jen1* gene of *S. cerevisie* was inserted into GS115 strain, resulting in the GJ strain (Table 1). Both strains, including the control (GS115), were used for the ^14^C lactate transport assay (Fig. 2 and Table 2). Nevertheless, no significant difference was found in the strain containing the *Jen1* gene and therefore this data is not shown.

**Table 2:** Vmax and Km of GS115 and GLS strains determined by using radiolabeled lactate

Strain	Vmax (μmol/s mg)	Km (µmolar)
GS115	11.68 ± 2.09	430.70 ± 105.00
GLS	7.54 ± 0.67	157.40 ± 25.46

The lactate transport by the GS115 and GS strains was assayed using radiolabeled lactate. Both GS and the wild-type strain (GS115) were incubated in the presence of ^14^C lactate, and the uptake capacity was further measured (Fig. 2). As can be seen in Fig. 2, the *P. pastoris* GS115 strain showed a higher velocity of transport, indicating that the GS strain rather increased the affinity for lactate. When Km and Vmax were calculated for all strains, these results were confirmed, where GS showed an increase of about threefold in affinity for lactate when compared with GS115 (Table 3).

**Table 3:** Kinetic parameters during fed-batch experiments at limited aerobic phase

Strain	Y_x/s_	Y_lac/s_	Y_ac/s_	Y_ara/s_	q_x_	q_lac_	q_ac_	q_ara_	r_x_	r_lac_	r_ac_	r_ara_
XL	0.180 ± 0.004	0.460 ± 0.004	0.008 ± 0.001	0.000 ± 0.000	0.020 ± 0.004	0.053 ± 0.012	0.001 ± 0.000	0.000 ± 0.000	0.117 ± 0.025	0.348 ± 0.073	0.006 ± 0.001	0.000 ± 0.000
GLJ	0.177 ± 0.004	0.470 ± 0.035	0.010 ± 0.005	0.000 ± 0.000	0.024 ± 0.003	0.063 ± 0.003	0.001 ± 0.000	0.000 ± 0.000	0.160 ± 0.054	0.413 ± 0.101	0.001 ± 0.004	0.000 ± 0.004
GLS	0.066 ± 0.004	0.673 ± 0.033	0.004 ± 0.002	0.001 ± 0.001	0.014 ± 0.002	0.146 ± 0.016	0.001 ± 0.000	0.000 ± 0.000	0.065 ± 0.005	0.673 ± 0.041	0.003 ± 0.001	0.001 ± 0.000

*Y* yield, *s* substrate, *x* biomass, *lac* lactate, *ac* acetate, *ara* arabitol, *Y* g/g, *q* g/g/h, *r* g/L/h

### Insertion of lactate-transporters results in increased lactate production in fed-batch fermentation

Once confirmed that *P. pastoris* could produce lactic acid using glycerol as a single carbon source and that the strains GS and GJ presented a higher affinity towards lactate when compared to the control strain, all constructed strains where evaluated for lactic acid production. To that end, fed-batch fermentation of the XL, GLJ and GLS strains was performed. Fed-batch was composed of two phases: the first one favoring biomass formation in aerobic conditions and the second initiated by the pH level change (>5) with a single pulse addition of 4 % glycerol and hypoxia conditions.

**Fig. 3:**
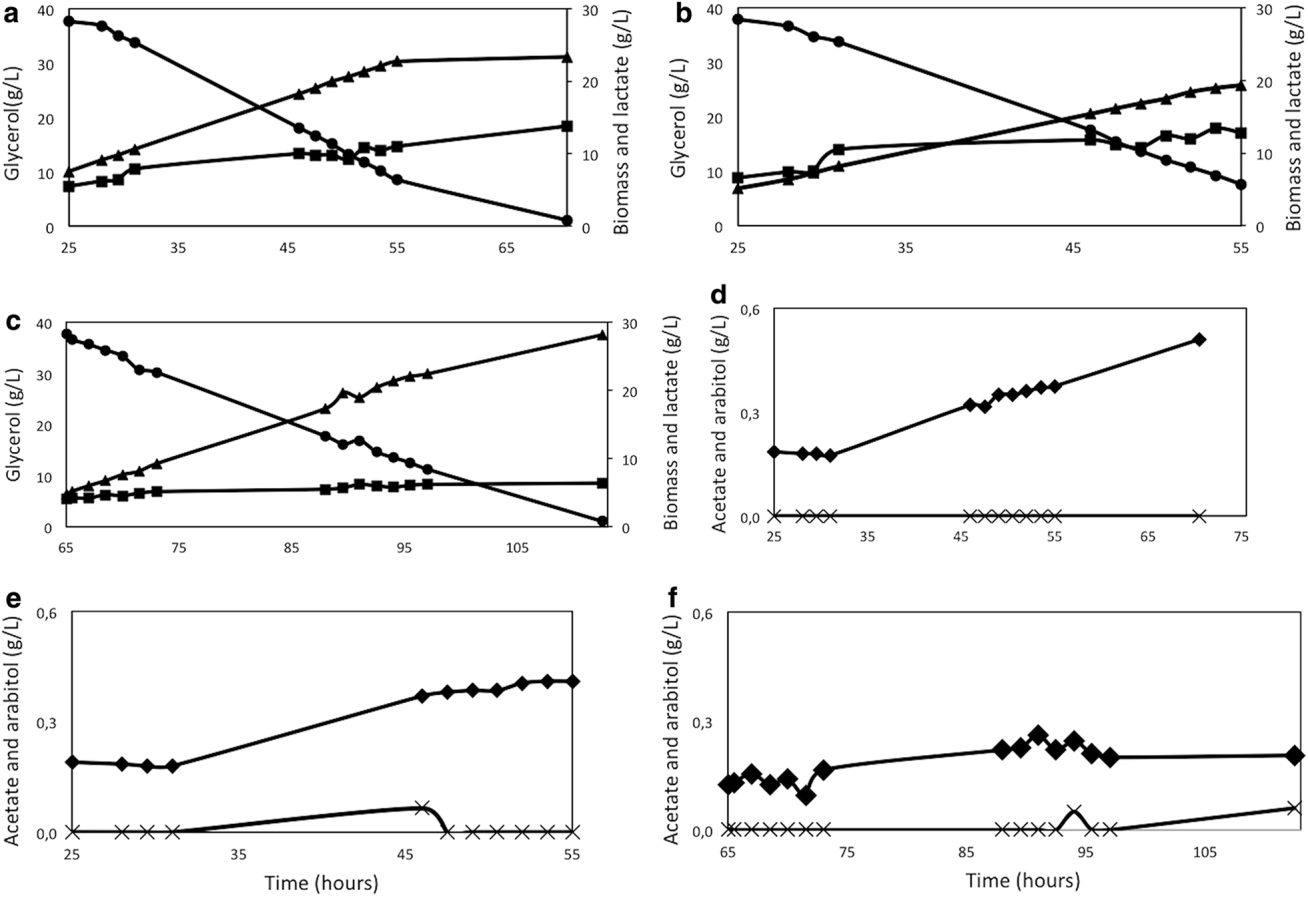
Fed-batch fermentation profile of the strains XL (**a** and **d**), GLJ (**b** and **e**) and GLS (**c** and **f**).** a**–**c** show the consumption of glycerol (*filled circle*) and the production of biomass (*filled square*) and lactate (*filled triangle*). The** c**–**f** show the formation of the products acetate (*filled diamond*) and arabitol (*cross*). Experiments were performed in triplicate. The figures show the profile of one of the fermentation within 10 % error

It can be observed that the GJ strain, although having shown higher affinity than the control strain towards lactate in the radiolabeled assay, presented a slightly higher lactate yield (2 %) when evaluated in a fed-batch experiment. On the other hand, GLS presented the highest lactate yield compared to XL and GLJ (46 and 43 % higher, respectively) (Fig. 3), as well as the highest lactate specific (0.126/h) and volumetric (0.673 g/L/h) productivity rates (Table 3). Moreover, at the fed-phase, GLS presented simultaneously the lowest biomass (approximately threefold) and acetate (2.5-fold) yields, showing that this strain is directing carbon towards lactate production instead of biomass formation. By-products such as acetate, arabitol and ethanol were also evaluated, and the kinetic parameters of the fed-phase showed that both XL and GLJ strains presented acetate approximately twofold higher yields, which is an indication of their lower lactate production (Table 3). For GLS, the increase in lactate yield was simultaneous with the reduction in arabitol, was close to zero in all strains and ethanol was never detected (data not shown).

## Discussion

### Limiting oxygen increases the production of l-lactic acid by *P. pastoris*

*Pichia pastoris* is a highly successful candidate for the production of heterologous protein (for review, see [21, 22]), however, few studies have been performed using the metabolic engineering of this microorganism [23–25]. Our study shows for the first time that genetically engineered *P. pastoris* strains containing LDH activity are able to produce lactic acid from glycerol. The use of glycerol by *P. pastoris* for the production of other biotechnological products such as phytase and recombinant human erythropoietin production has been previously reported [15, 26], making this organism a potential route for the conversion of crude glycerol into products of biotechnological interest. Introduction of LDH activity from *B. taurus* resulted in a lactate yield per consumed substrate 52 % higher than what has been previously found for the introduction of the same gene in *S. cerevisiae* (Y_lac/s_ 0.155 g/g) using glucose as substrate [18]. Furthermore, lactate production could be further improved by restricting oxygen limitation in the bioreactor [27, 28]. Our results suggest that a lower dissolved oxygen supply can provide higher production of heterologous lactic acid, which can be an effect of both increased yeast fermentation capacity and up regulation of the pGAP promoter. In a previous study, evaluation of heterologous production of a Fab fragment under the control of a pGAP promoter and limited oxygen conditions (8.39 and 5.87 %) showed that biomass yield decreased twofold while heterologous protein production increased by 2.5-fold [29]. It has been recently evaluated whether oxygen transfer affects protein production under the control of the pGAP by *P. pastoris*. It was observed that 20 % air saturation showed the highest volumetric activity of glucose isomerase when compared to 15 % DO [30]. Although we have not evaluated the enzyme activity in different aeration conditions, our result indicates that limiting oxygen availability pushes the yeast metabolism towards the fermentative pathway.

### New putative lactate transporter from *P. pastoris*

Since the identification of *JEN1* coding for the lactate transporter in *S. cerevisiae* [19], it was reported that the Jen1p transporter was able to reconstitute the lactate permease activity in *P. pastoris* [20]. Moreover, in the previous study the background activity of the control strain (KM71H) could not be measured and the kinetic parameters Vmax (2.15 ± 0.14 nmol of lactic acid/s mg of dry weight) and Km (0.54 ± 0.08 mM) could only be determined for the recombinant strain producing the Jen1p transporter [20]. In the present study we did not see a significant difference between the control strain and the one over producing Jen1P, despite a tendency of the GJ strain has a greater affinity to lactate. Regarding the strains overproducing the putative lactate transporter from *P. pastoris*, GS strain, it showed a threefold higher affinity to lactate compared with control strain.

### GLS strain provides the highest lactate secretion in fed-batch

Among tested strains and fermentation modes, the best performance was achieved by GLS in oxygen limited fed-batch fermentation. Production of lactic acid using glycerol has been reported using other organisms such as the Gram-positive lactic acid bacteria *E. faecalis* [3]. This later study used fed batch with 30 g/L of glycerol coupled with 22 g/L of acetic acid as substrate and they achieved a lactic acid volumetric productivity rate 7 % higher than what was achieved in our study using the GLS strain. Another study evaluated the production of lactic acid using the fungi *Rhizopus oryzae* in a batch fermentation with 40 g/L of crude glycerol plus either inorganic nutrients of lucern green juice [7]. In both cases the total amount of lactic acid at the end of the fermentation was approximately 12 % lower than what has been found in the present study. Although these studies have shown the production of lactic acid using either crude or pure glycerol, they did not use engineered strains like the one presented here.

It has been previously reported that the cost of bio-production of polylactic acid depends on the substrate used in its process [31]. Crude glycerol is obtained as a residue from the conversion of vegetable oils into biodiesel. It is estimated that for every 9 kg of biodiesel produced, 1 kg of crude glycerol is generated [32]. Thus, for its high availability and low cost, crude glycerol is an excellent candidate for high-added-value chemical production including lactic acid [4, 33, 34, 35]. We have developed a novel *P. pastoris* engineered strain that produces lactic acid using pure glycerol as unique carbon source. Our strain GLS, which has achieved the highest l-lactic acid yield of 0.7 g/g, shows a result close to the maximum theoretical yield of 1.0 g/g.

## Conclusions

This study has shown for the first time that genetically engineered *P. pastoris* strains are able to produce L-lactic acid, making this organism a potential biocatalyst for the conversion of crude glycerol into products of biotechnological interest. Another novelty of this study is the identification of a putative lactate transporter in *P. pastoris.* Two genetically modified strains carrying lactate transporters were developed in order to improve the secretion of L-lactic acid. Both strains named GLJ and GLS showed higher affinity towards lactate when compared to the control strain. Fed-batch fermentation processes fed with 40 g/L glycerol showed that GLS presented an increase of 46 % in lactate yield compared to the control strain and 43 % higher than GLJ. The lactate volumetric and specific productivity rates were higher in GLS with a concomitant decrease in biomass and lactate yields of approximately 60 % each were also observed.

## Methods

### Plasmids and strains

The plasmids and strains used in this study are listed in Table 1. The bacterial strains were grown at 37 °C in Luria broth medium (0.5 % yeast extract, 1 % peptone and 1 % sodium chloride), and the yeast strains were grown at 30 °C in YPD (0.5 % yeast extract, 1 % peptone and 2 % dextrose). When required, the medium were supplemented with the appropriate antibiotics: ampicillin for *E. coli* cultivation (100 µg/mL) and zeocin for *P. pastoris* cultivation (100 µg/mL).

### Strain construction

In order to produce lactic acid, the plasmid pGAPZB (Life Technologies, Carlsbad, CA, USA) containing the *Bos Taurus ldh* codon-optimized gene was linearized with *Avr*II restriction enzyme (Life technology, San Diego, CA, USA) and integrated by homologous recombination into the *P. pastoris* X-33 chromosomal DNA. The resulting plasmid named pGAP-LDH was synthesized by Genome Company (Madison, WI, USA). The integration was confirmed by resistant clones selected in zeocin (100 μg/mL). The resulting strain was named XL (Table 1). In order to evaluate the influence of the lactate transporter in *P. pastoris* strains producing LDH, the pPICPGKGFP plasmid (derived from pPIC9 k-Life technology) was used. pPICPGKGFP containing either the codon-optimized gene encoding for the lactic acid transporter *Jen1p* from *S. cerevisiae* or the codon-optimized gene encoding for the putative transporter of *P. pastoris Pas* were developed, resulting in the plasmids pPGK-JEN and pPGK-PAS, respectively (Table 1). The primers used for the construction of pPGK-JEN were JEN-F 5′ATTCGCGGCCGCATGTCGTCGTCAATTACA3’ and JEN-R5’ TTAAACGGTCTCAATATGCTGAATTCATC3’ (*Not*RI and *Eco*RI restriction sites underlined, respectively), and the primers used for the construction of pPGK-PAS were PAS-F5’ ATTCGCGGCCGCATGTCGCATTCAATCCATT3’ and PAS-R5’ TTACTTATTTCCTTCAAAAGCCGAATTCATC3′ (*Not*RI and *Eco*RI restriction sites underlined, respectively). The plasmids pPGK-JEN and pPGK-PAS were integrated into the GS115 (*his*4) strains using the restriction enzyme *Bgl*II (Life technology, San Diego, CA, USA). Positive clones were selected in YNB solid medium without amino acids, and the resulting strains were respectively named GJ and GS. Next, GJ and GS strains were transformed with pGAP-LDH, resulting in the strains GLJ and GLS, respectively (Table 1).

### Enzyme activities

Enzyme assays were carried out as described previously with modifications [36]. Briefly, a primary inoculum culture was prepared in YPD medium, with zeocin (100 μg/mL), and maintained at 30 °C and 180 rpm overnight. Cells were harvested, re-inoculated in a new flask, and grown in a shaker at 30 °C until the exponential phase. After centrifugation, cells were ressuspended in Yeast Protein Extraction Reagent (Y-Per, Life Technologies) for 10 min. The reaction was assembled with cellular extract, 10 µL; NADH, 8 µL; 50 mM phosphate buffer (pH 8.0), 800 µL, and ultra-pure water for a 1 mL final volume. After 150 s, pyruvate 40 µL was added and the reaction was completed in 300 s. Then, LDH activity was determined at 30 °C through the absorbance reduction at 340 nm caused by oxidation of NADH cofactor after pyruvate addition as substrate. The unit of enzyme activity was defined as the amount of enzyme necessary to oxidize 1 μmol NADH per minute.

## ^14^C-lactate transport in the strains containing Jen1p and PAS

Radiolabelled lactic acid uptake was determined as described previously [20] with modifications. Briefly, a primary inoculum culture for GS115 (control), GJ and GS strains were prepared in YPD medium and grown until the exponential phase of growth at 30 °C and 180 rpm. Cells were harvested, re-inoculated in YNB medium with 0.5 % l-(+)-lactic acid (Sigma Aldrich Co., USA) for glucose starvation during 4 h in shake at 30 °C. The inoculums were centrifuged at 12,000*g* for 5 min and washed twice with ice-cold water. Cells were ressuspended in KH_2_PO_4_ 1 M buffer, pH 5.0 and kept at room temperature during experiment. A mix containing 1 uL of [U-14C] lactic acid (sodium salt; 106.9 mCi/mmol [3.955 GBq/mmol], Perkin Elmer Life Science) and 9 uL of different concentrations of non-radiolabeled lactic acid (Sigma Aldrich Co., USA) was added to 40 µL of washed cells. After 10 s of incubation, the uptake reaction was quenched by the addition of 1 mL of ice-cold water. Cells were quickly filtered through a nitrocellulose filter 0.45 µm (Sartorius, Gottingen, Germany) linked to a vacuum filter system. After extensive washes and removal of the non-incorporated radiolabelled lactic acid, the membranes containing the washed cells were transferred to a scintillation tube containing 3 mL of scintillation fluid (ScientiSafe; Thermo Fisher Scientific, Whatman, MA, USA). The intracellular radioactivity was measured in the Packard Tri-Carb 2200 CA liquid scintillation spectrophotometer with disintegrations per minute correction. To determine the non-specific 14C adsorption, the labeled lactic acid was added at zero time after the addition of ice-cold water. To determine the transport kinetics that best fit with the experimental values of initial lactate uptake rates and to estimate the kinetic parameters, a computer-assisted non-linear regression analysis was used (GraphPAD software). All experiments were repeated at least three times and the data reported are the average values. For comparison between strains, an ANOVA Tukey Test was used.

### Medium for batch and fed-batch

In batch and fed-batch experiments, a defined medium was utilized as previously described with modifications [37]. The composition of the medium (per liter) was: 20 or 40 g glycerol·1H_2_O, 1.8 g C_6_H_8_O_7_, 0.02 g CaCl_2_·2H_2_O, 12.6 g (NH_4_)2HPO_4_, 0.5 g MgSO_4_·7H_2_O, 0.9 g KCl, and 4.35 mL PTM1 trace salts stock solution, and pH was set to 5.0 with 25 % HCl. PTM1 trace salts stock solution (per liter) was composed by: 6.0 g CuSO_4_·5H_2_O, 0.08 g NaI, 3.0 g MnSO_4_·H_2_O, 0.2 g Na_2_MoO_4_·2H_2_O, 0.02 g H_3_BO_3_, 0.5 g CoCl_2_, 20.0 g ZnCl_2_, 14.3 g FeSO_4_ and 5.0 mL H_2_SO_4_ (95–98 %), 0.4 g biotin. 0.04 g histidin was supplemented for the strain GS115.

### Fermentation parameters for batch and fed-batch

For batch experiments, the pre-inoculum culture was prepared with 20 g/L glycerol in 100 mL defined medium in a 1 L flask, and it grew for approximately 48 h at 30 °C and 200 rpm. This pre-culture was then used to inoculate 500 ml defined medium at an initial OD_600nm_ of 2.0 into 1 L Infors HT fermentor (Infors HT, Bottmingen, Switzerland). The glycerol concentration in the defined medium for batch experiments was 40 g/L. To evaluate the production of lactic acid in XL, a batch experiment was performed at the following conditions: 30 °C, 500 rpm, air flow 0.05 vvm, dissolved oxygen at either 5 or 3 % and pH 5 controlled with 5 M NH_4_OH. To evaluate the correlation between amount of dissolved oxygen and lactic acid production, a batch fermentation in cascade mode was performed at the following conditions: pH 5 controlled with 5 M NH_4_OH, 30 °C, and dissolved oxygen at 3 or 5 % measured by a sterilized electrode (Mettler-Toledo, Moburn, MA, USA), maintained by computational adjustment of the rotation speed (minimum 350/set 500/maximum 900 rpm) and air flow (minimum 0.05/set 0.05/maximum 0.5 vvm). The batch fermentations were run for 30 h. Samples were collected every 90 min and centrifuged at 12,000*g* for 2 min, then the supernatant was stored at −20 °C for HPLC analysis. For fed-batch, the pre-inoculum was prepared as described above. The fed-batch fermentations were performed with an initial glycerol concentration of 20 g/L at the following conditions: 30 °C, 500 rpm, air flow 0.05 vvm, dissolved oxygen at 30 % and pH 5 controlled with 5 M NH_4_OH. When pH went above 5, the feeding step was initiated with the addition of 40 g/L glycerol in a single pulse. Samples were collected every 90 min and centrifuged at 12,000*g* for 2 min. The supernatant was stored at −20 °C for HPLC analysis.

### Substrate consumption, lactic acid, biomass and by-product formation

Acetic acid, ethanol, arabitol, lactic acid and glycerol were quantified using a Hewlett-Packard High-performance liquid chromatograph (HPLC) (Shimadzu, Kyoto, Japan) equipped with UV (210-nm) and refractive index detectors. A pre-column Guard Column SCR (H) (50 mm × 4 mm id) with stationary phase sulphonated styrene–divinylbenzene copolymer resin was used. The chromatography was performed using a Shim-pack SCR-101H (Shimadzu) (300 mm x 7.9 mm id) column equilibrated at 60 °C with 5 mM H_2_SO_4_ as the mobile phase at flow rate of 0.6 mL/min, and an injection volume of 20 µL. The run was 26 min long. For the analysis of biomass, dry cell weight (DCW) samples were collected for OD_600nm_ measurement and the same sample was dried then weighed. OD_600nm_ was converted to DCW (g/L) using the appropriate calibration curve; 1 unit of OD_600nm_ corresponded to 0.390 g DCW/L.
